# Genetically engineered senescence-resistant human mesenchymal progenitor cells promote spinal cord injury repair

**DOI:** 10.1093/lifemedi/lnaf038

**Published:** 2025-11-29

**Authors:** Taixin Ning, Jinghui Lei, Xiaoyu Jiang, Shuhui Sun, Fangshuo Zheng, Qian Zhao, Shuai Ma, Weiqi Zhang, Jing Qu, Guang-Hui Liu, Si Wang

**Affiliations:** Advanced Innovation Center for Human Brain Protection, National Clinical Research Center for Geriatric Disorders, Aging Translational Medicine Center, Beijing Municipal Geriatric Medical Research Center, Beijing Key Laboratory of Environment and Aging, Xuanwu Hospital, Capital Medical University, Beijing 100053, China; Advanced Innovation Center for Human Brain Protection, National Clinical Research Center for Geriatric Disorders, Aging Translational Medicine Center, Beijing Municipal Geriatric Medical Research Center, Beijing Key Laboratory of Environment and Aging, Xuanwu Hospital, Capital Medical University, Beijing 100053, China; State Key Laboratory of Organ Regeneration and Reconstruction, Institute of Zoology, Chinese Academy of Sciences, Beijing 100101, China; University of Chinese Academy of Sciences, Beijing 100049, China; Beijing Institute of Heart Lung and Blood Vessel Diseases, Beijing Anzhen Hospital, Capital Medical University, Beijing 100029, China; Chongqing Fifth People’s Hospital, Chongqing 400060, China; Advanced Innovation Center for Human Brain Protection, National Clinical Research Center for Geriatric Disorders, Aging Translational Medicine Center, Beijing Municipal Geriatric Medical Research Center, Beijing Key Laboratory of Environment and Aging, Xuanwu Hospital, Capital Medical University, Beijing 100053, China; State Key Laboratory of Organ Regeneration and Reconstruction, Institute of Zoology, Chinese Academy of Sciences, Beijing 100101, China; University of Chinese Academy of Sciences, Beijing 100049, China; Beijing Institute for Stem Cell and Regenerative Medicine, Beijing 100101, China; Aging Biomarker Consortium (ABC), Beijing 100101, China; University of Chinese Academy of Sciences, Beijing 100049, China; Aging Biomarker Consortium (ABC), Beijing 100101, China; Beijing Institute of Genomics, China National Center for Bioinformation, Chinese Academy of Sciences, Beijing 100101, China; Sino-Danish College, Sino-Danish Centre for Education and Research, University of Chinese Academy of Sciences, Beijing 100049, China; State Key Laboratory of Organ Regeneration and Reconstruction, Institute of Zoology, Chinese Academy of Sciences, Beijing 100101, China; University of Chinese Academy of Sciences, Beijing 100049, China; Beijing Institute of Heart Lung and Blood Vessel Diseases, Beijing Anzhen Hospital, Capital Medical University, Beijing 100029, China; Beijing Institute for Stem Cell and Regenerative Medicine, Beijing 100101, China; Aging Biomarker Consortium (ABC), Beijing 100101, China; Human Organ Physiopathology Emulation System, Institute of Zoology, Chinese Academy of Sciences, Beijing 100101, China; State Key Laboratory of Organ Regeneration and Reconstruction, Institute of Zoology, Chinese Academy of Sciences, Beijing 100101, China; University of Chinese Academy of Sciences, Beijing 100049, China; Beijing Institute for Stem Cell and Regenerative Medicine, Beijing 100101, China; Aging Biomarker Consortium (ABC), Beijing 100101, China; Human Organ Physiopathology Emulation System, Institute of Zoology, Chinese Academy of Sciences, Beijing 100101, China; Advanced Innovation Center for Human Brain Protection, National Clinical Research Center for Geriatric Disorders, Aging Translational Medicine Center, Beijing Municipal Geriatric Medical Research Center, Beijing Key Laboratory of Environment and Aging, Xuanwu Hospital, Capital Medical University, Beijing 100053, China; Aging Biomarker Consortium (ABC), Beijing 100101, China

**Keywords:** FOXO3, SRC, spinal cord injury, cell therapy, neuroprotection

## Abstract

Spinal cord injury (SCI) is a devastating condition affecting the central nervous system, often leading to persistent neurological dysfunction. While mesenchymal progenitor cells (MPCs) hold considerable promise for treating various disorders, their application in SCI repair remains hampered by challenges such as poor efficacy and safety concerns. In this study, we developed genetically engineered human MPCs with enhanced resistance to senescence and stress—termed senescence- and stress-resistant cells (SRCs)—and systematically evaluated their therapeutic potential and mechanisms in SCI repair. Intramedullary implantation of SRCs improved functional recovery after SCI. Mechanistically, SRCs exerted therapeutic effects through a dual approach: by mitigating neuronal and axonal loss while stimulating endogenous neuroregeneration, and by suppressing neuroinflammation while modulating astrocyte distribution to restrict lesion expansion. Importantly, we identified exosomes derived from SRCs as key mediators of these reparative effects. Our findings provide comprehensive insights into the therapeutic role of engineered SRCs in SCI repair, delineating both direct cellular and exosome-mediated mechanisms, thus providing experimental support for future clinical translation.

## Introduction

Spinal cord injury (SCI) is a severe traumatic disorder of the central nervous system (CNS) that causes profound motor and sensory dysfunction, greatly diminishes the quality of life, and imposes a heavy burden on patients [1, 2]. Epidemiological data indicate that as of 2019, the global prevalence of SCI had reached 20.6 million cases, with an annual increase of approximately 900,000 new cases [3]. Despite notable advances in neuroregeneration research, clinically effective therapeutic strategies to facilitate functional recovery following SCI remain limited. This unmet medical need underscores the urgency of developing innovative repair strategies for SCI.

Stem cells represent a promising tool for regenerative medicine [4–7]. Among them, mesenchymal progenitor cells (MPCs) have attracted considerable interest due to their self-renewal capacity, multilineage differentiation potential, and immunomodulatory properties [8, 9]. However, the clinical translation of stem/progenitor cell-based therapies faces major obstacles, including cellular senescence—characterized by reduced proliferation and differentiation capacity after prolonged *in vitro* culture—as well as poor survival and low retention rates post-transplantation [10–12]. To overcome these limitations, we employed gene-editing techniques to generate *FOXO3*-engineered senescence- and stress-resistant human MPCs (SRCs), which demonstrate enhanced resistance to senescence and stress while being protected against malignant transformation [13–15]. Previous studies have demonstrated that SRC intervention not only delays natural aging in non-human primates but also exerts therapeutic effects in models of ischemic diseases [14, 16, 17]. Nevertheless, it is unclear whether SRCs can exert therapeutic effects within the adverse post-SCI microenvironment—characterized by a robust inflammatory response, glial scar formation, and neuronal cell death—which collectively pose a severe barrier to neuroregeneration [18, 19].

In this study, we used a mouse model of SCI to systematically evaluate the therapeutic effects of intramedullary SRC implantation on neural repair. We found that SRC transplantation improved motor function in SCI mice. Furthermore, SRCs attenuated local inflammation and regulated the spatial distribution of astrocytes, thereby limiting injury expansion. Importantly, SRCs exerted neuroprotective effects by reducing neuronal and axonal loss and enhancing endogenous neuroregeneration. Notably, exosomes derived from SRCs (SRC-Exo) partially recapitulated the benefits of cell implantation, providing important evidence for the development of cell-free stem cell therapies.

## Results

### Generation and characterization of SRCs

**Figure 1.**
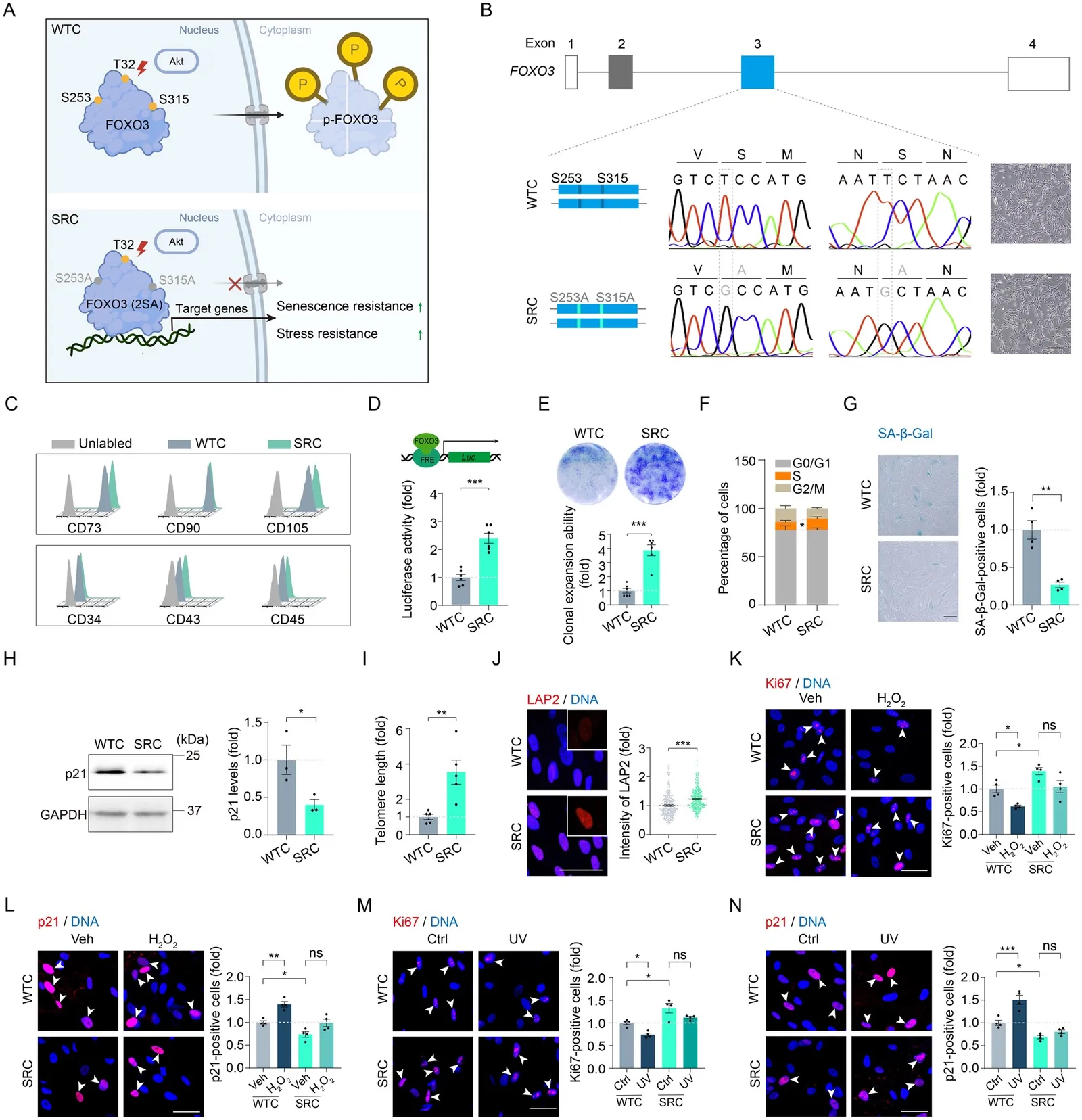
Generation and characterization of SRCs. (A) Diagram showing that the engineered FOXO3 cannot be phosphorylated at serines 253 and 315, leading to its sustained nuclear localization and constitutive transcriptional activity. (B) DNA sequencing showing the bases conversion in WTCs and SRCs (left) and cell morphology of WTCs and SRCs (right). (C) Fluorescence-activated cell sorting (FACS) analysis of the expression of MPCs-specific markers (CD73, CD90, and CD105) and MPCs-irrelevant markers (CD34, CD43 and CD45). (D) FOXO3 transcriptional activity assays in WTCs and SRCs with pGL3-FHRE vector infection. (E) Clonal expansion assays of WTCs and SRCs. (F) Cell cycle analysis of WTCs and SRCs. (G) SA-β-Gal staining of WTCs and SRCs. (H) Western blot analysis of p21 of WTCs and SRCs. (I) Telomere length assays of WTCs and SRCs. (J) Immunostaining of LAP2 of WTCs and SRCs. (K) Immunostaining of Ki67 of WTCs and SRCs after vehicle (Veh) or H_2_O_2_ treatment. (L) Immunostaining of p21 of WTCs and SRCs after Veh or H_2_O_2_ treatment. (M) Immunostaining of Ki67 of WTCs and SRCs in Control or UV treatment groups. (N) Immunostaining of p21 of WTCs and SRCs in Control or UV treatment groups. Quantitative data are shown as means ± SEMs. The arrowheads indicate positive cells in (K)–(N). *n =* 3–6 biological samples per group in (D)–(I) and (K)–(N); *n* > 200 cells obtained from per group in (J). Two-sided Student’s *t* test or Wilcoxon rank-sum test was performed in (D)–(J); one-way ANOVA followed by Dunnett’s multiple comparisons test was performed in (K)–(N). Scale bar, 50 μm in (B), (G) and (J)–(N).

Using gene-editing technology, we targeted two critical phosphorylation sites (S253 and S315) of FOXO3 for modification. This approach effectively blocked the phosphorylation-dependent nuclear export of FOXO3, thereby enhancing its nuclear stability and transcriptional activity [13] (Fig. 1A). Next, we applied a directed differentiation technique to generate *FOXO3* gene-engineered SRCs (Fig. 1A and 1B). Notably, SRCs exhibited a specific marker profile akin to wild-type MPCs (WTCs) (Fig. 1C). Furthermore, a luciferase reporter assay confirmed that the transcriptional activity of FOXO3 in SRCs was higher than in WTCs (Fig. 1D).

In terms of functional characterization, we found that compared to WTCs, SRCs exhibited an enhanced proliferative capacity, as evidenced by increased clonal expansion ability and a higher proportion of cells in the S phase (Fig. 1E and 1F). Additionally, the activity of senescence-associated β-galactosidase (SA-β-Gal) and the expression levels of the cell cycle arrest protein p21 were reduced in SRCs (Fig. 1G and 1H). Moreover, SRCs exhibited longer telomeres compared to WTCs (Fig. 1I). Immunostaining analysis demonstrated increased expression of lamina-associated polypeptide 2 (LAP2) in SRCs (Fig. 1J), indicating enhanced nuclear lamina integrity [20]. Collectively, these results validate the senescence-resistant properties of SRCs.

To assess the stress resistance of SRCs, we subjected them to H_2_O_2_-induced oxidative stress and UV-induced DNA damage. Under both conditions, SRCs demonstrated a higher percentage of Ki67-positive proliferating cells and a lower proportion of p21-positive senescent cells compared to WTCs (Fig. 1K–N). These results confirm that SRCs are resistant to ­senescence triggered by oxidative and genotoxic stress, providing key experimental support for their resilience in adverse microenvironments.

### Construction of a SCI mouse model and intramedullary tracing of MPCs

**Figure 2.**
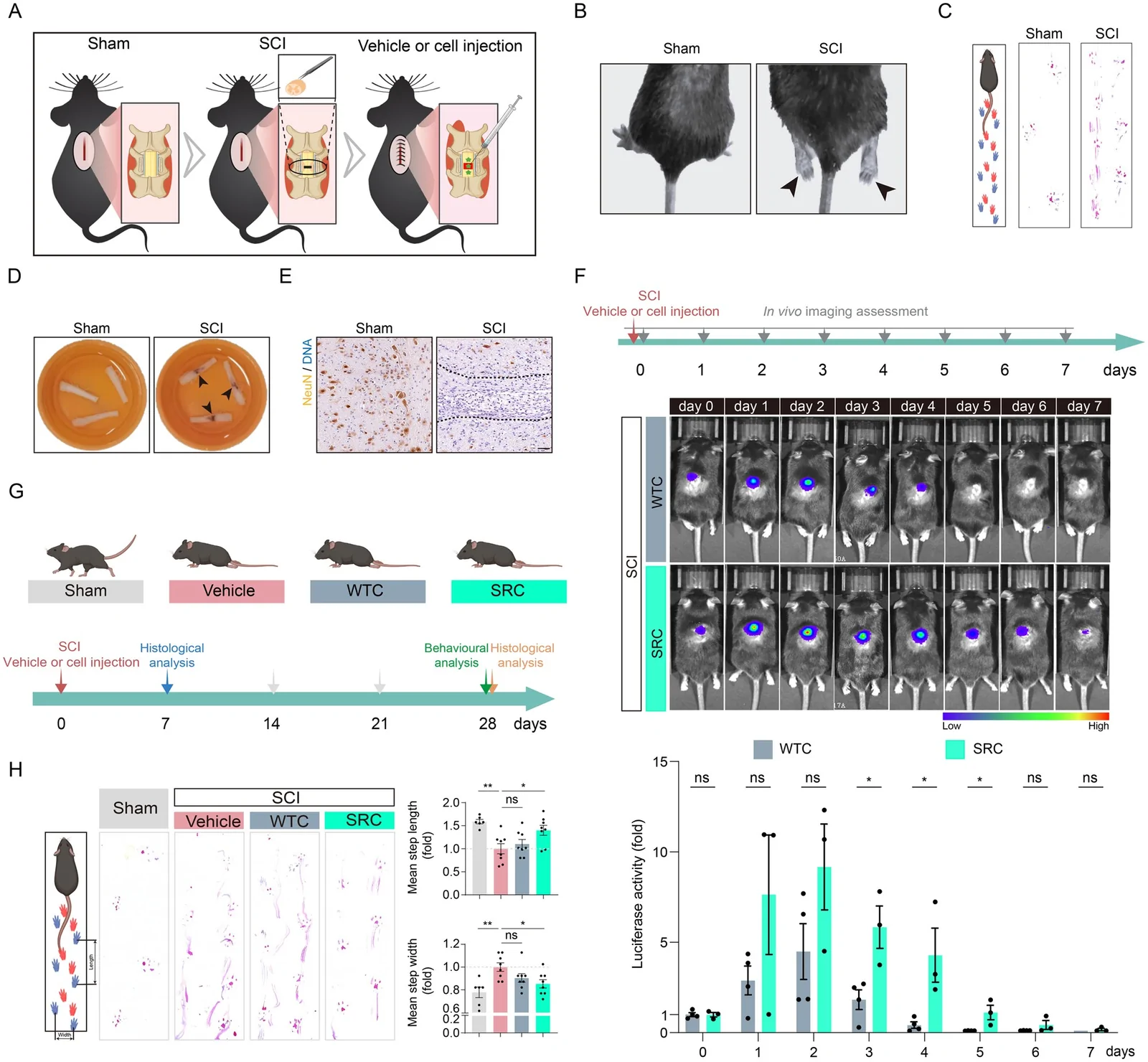
SRC treatment promotes functional recovery after SCI.(A) Schematic representation of the SCI mouse model and intramedullary microinjection. (B) Representative images of motor characteristics in sham and SCI mice. (C) Representative images of footprint in sham and SCI mice. (D) Representative images of spinal cord tissues in sham and SCI mice. (E) Immunostaining of NeuN in spinal cord of sham and SCI mice. (F) Retention evaluation of WTCs and SRCs using *in vivo* bioluminescence imaging of luciferase-labeled cells. (G) Schematic representation of the experimental design, including animal grouping, the timeline of cell injection and subsequent functional and histological assessments. (H) Footprint analysis in mice across indicated groups. Quantitative data are shown as means ± SEMs. The arrowheads indicate the locomotor characteristics of the hindlimbs in (B) and injury site characteristics in (D) after SCI. Dashed lines indicate the injury border in (E). *n =* 3–4 biological samples per group and Two-sided Student’s *t* test or Wilcoxon rank-sum test was performed in (F). *n =* 6–8 biological samples per group and one-way ANOVA followed by Dunnett’s multiple comparisons test was performed in (H). Scale bar, 50 μm in (E).

To mimic clinical SCI pathology, we generated a murine contusion model at the T10 spinal segment [21]. This model successfully recapitulated key human SCI features, including motor deficits and neural tissue damage (Fig. 2A). Specifically, SCI mice displayed immediate hindlimb retraction and loss of support after surgery, in contrast to sham-operated animals (Fig. 2B). Gait analysis at 28 days further revealed pronounced abnormalities—including hindlimb dragging, shortened stride length, and increased stride width—confirming persistent motor impairment (Fig. 2C) [22]. Concurrently, anatomical and histopathological examination showed typical scar formation and near-total neuronal loss at the injury epicenter (Fig. 2D and 2E). Together, these findings demonstrate the successful establishment of a clinically relevant SCI model.

To precisely evaluate the therapeutic effects of SRCs, we developed an intramedullary cell injection and tracing protocol. Subsequently, 1 × 10^5^ luciferase-labeled WTCs and SRCs were precisely implanted into the injury epicenter [17]. *In vivo* imaging showed that on day 0, bioluminescent intensities from both cell groups were closely matched, indicating comparable initial *in vivo* states (Fig. 2F). As the observation period extended, both groups exhibited a rapid rise and gradual decline in bioluminescent signals (Fig. 2F). Throughout the 7-day observation period, the bioluminescent signal in the SRC group remained consistently higher than that in the WTC group (Fig. 2F). These findings suggest that under the pathological conditions of SCI, genetically modified SRCs exhibit enhanced proliferative activity, survival capacity, and tissue retention properties.

### SRC intramedullary implantation promotes functional recovery after SCI

To evaluate the therapeutic potential of SRCs in SCI repair, we first performed behavioral analyses of motor function at 28 days post-implantation (Fig. 2G). Quantitative gait analysis revealed that vehicle-treated SCI mice developed pronounced deficits, including a decreased average stride length and an increased stride width compared to the sham group—findings consistent with established SCI pathology [22]. In contrast, SRC-treated mice exhibited gait improvement, characterized by increased stride length and reduced stride width (Fig. 2H). These results demonstrate that intramedullary implantation of SRCs promotes the recovery of hindlimb motor coordination after SCI.

### SRC treatment alleviates the initial inflammatory microenvironment following SCI

**Figure 3.**
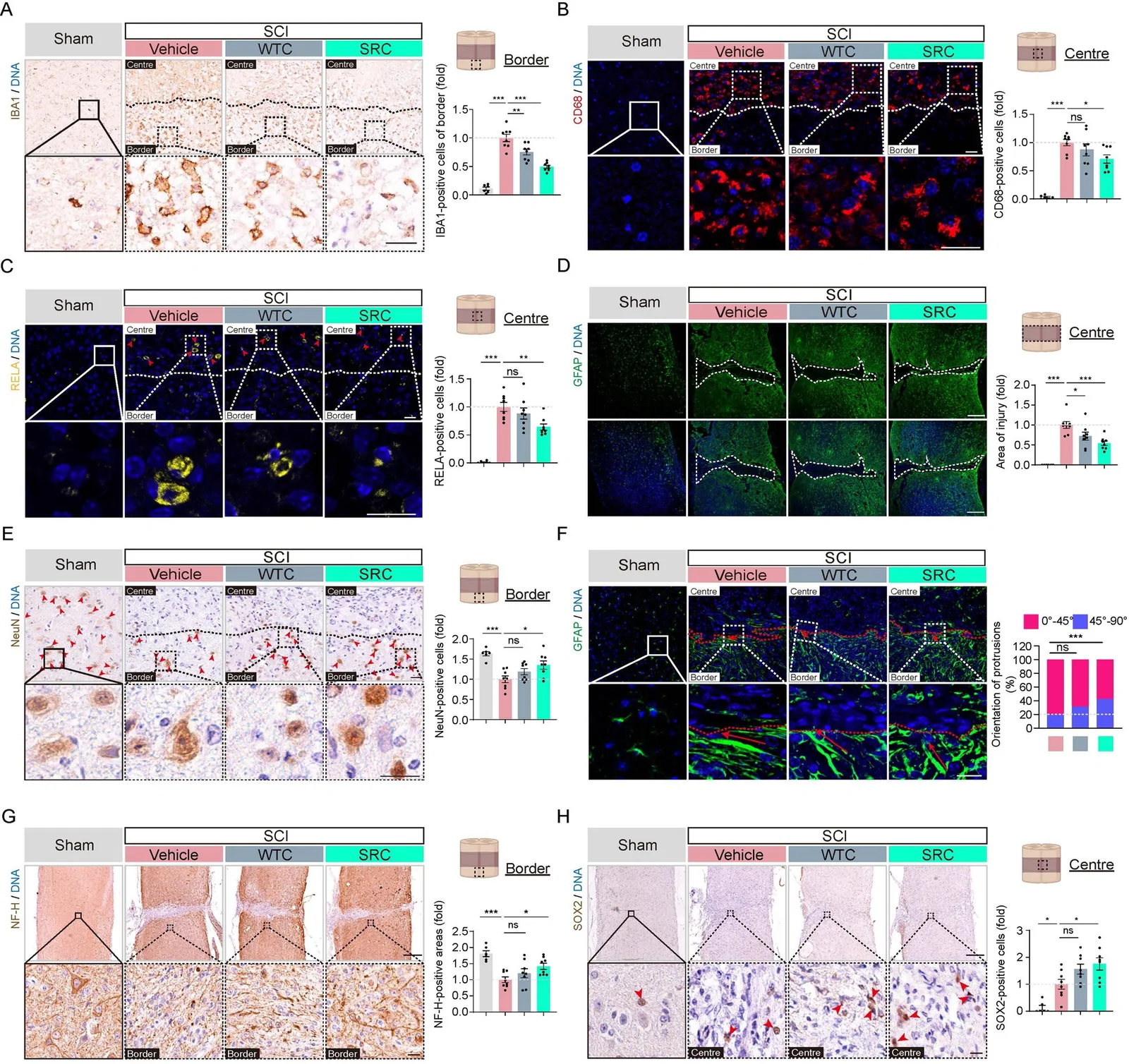
SRCs reduce neuroinflammation and promote neuroprotection following SCI.(A) Immunostaining analysis of IBA1 in spinal cord of mice across indicated groups. (B) Immunostaining analysis of CD68 in spinal cord of mice across indicated groups. (C) Immunostaining analysis of RELA in spinal cord of mice across indicated groups. (D) Immunostaining analysis of GFAP in spinal cord of mice across indicated groups. White dashed lines outline the injury epicenter. (E) Immunostaining analysis of NeuN in spinal cord of mice across indicated groups. (F) Immunostaining analysis of GFAP in spinal cord of mice across indicated groups. Red dashed lines indicate the injury borders and the solid red arrows indicate the direction of the astrocyte protrusion. (G) Immunostaining analysis of NF-H in spinal cord of mice across indicated groups. (H) Immunostaining analysis of SOX2 in spinal cord of mice across indicated groups. Quantitative data are shown as means ± SEMs. *n =* 6–8 biological samples per group in (A)–(H). One-way ANOVA followed by Dunnett’s multiple comparisons test was performed in (A)–(H). Dashed lines indicate the injury border in (A)–(H); solid and dashed squares indicate the zoom-in areas in (A)–(C), (E)–(H). Scale bar, 20 μm in (A)–(C), (E), (F), lower panel of (G) and (H); 200 μm in (D), upper panel of (G) and (H).

Given that neuroinflammation is a well-established early feature of SCI, with the period of 0–7 days post-injury characterized by robust immune cell infiltration and pro-inflammatory cytokine release [23], we sought to determine whether SRC implantation modulates this inflammatory microenvironment. We performed a histological analysis of spinal cord tissues at 7 days post-injury (Fig. 2G). Immunofluorescence staining revealed a pronounced accumulation of IBA1^+^ microglia at the lesion border in vehicle-treated mice (Fig. 3A), consistent with classic SCI pathology [23]. In contrast, SRC-treated mice exhibited a reduced number of IBA1^+^ microglia at this site (Fig. 3A), indicating that SRC implantation mitigated the local neuroinflammatory response. Notably, CD68, a marker of activated macrophages/microglia [24–26], was extensively expressed in the injury epicenter but not in the microglial-enriched border region; this pattern is likely attributable to infiltrating peripheral macrophages following blood-spinal cord barrier (BSCB) disruption [27]. SRC implantation reduced the number of CD68^+^ cells in the epicenter (Fig. 3B), suggesting enhanced BSCB integrity and diminished infiltration of pro-inflammatory immune cells [28]. At the molecular level, immunofluorescence analysis showed downregulation of RELA (a core subunit of NF-κB [29, 30]) in the SRC group compared to the vehicle group (Fig. 3C). This implies that SRCs may alleviate neuroinflammation partly by suppressing the NF-κB signaling pathway. Collectively, these findings demonstrate that SRC treatment ameliorates the inflammatory microenvironment post-SCI through multiple mechanisms, including inhibiting over-activation of microglia/macrophages and modulating key inflammatory signaling pathways such as the NF-κB pathway.

### SRCs promote neuroprotection and foster a pro-regenerative niche after SCI

To further elucidate the long-term impact of SRCs on tissue repair, we analyzed the injury site at 28 days post-SCI. Our findings revealed that SRC implantation reduced the area of the injury epicenter (Fig. 3D), indicating a role in limiting lesion expansion. This structural preservation was accompanied by enhanced neuronal survival. SRCs demonstrated a region-specific neuroprotective effect, increasing the number of preserved neurons at the vulnerable injury border, although neurons within the lesion epicenter were not rescued (Fig. 3E). As neurons are the primary functional units of the nervous system [31], their protection at the border zone likely constitutes a critical prerequisite for functional recovery.

Beyond conferring structural and neuronal protection, SRCs actively promoted a pro-regenerative microenvironment. They induced a morphological polarization of astrocytes, shifting their alignment from parallel to a radial configuration perpendicular to the lesion (Fig. 3F)—a pattern demonstrated previously to facilitate axonal regeneration [32, 33]. Consistent with this supportive restructuring, we observed a marked increase in neurite density within the injury area of SRC-treated mice (Fig. 3G), providing a structural basis for the observed functional improvement [34].

Furthermore, the reparative process involved the mobilization of endogenous neural stem/progenitor cells, as evidenced by an increased accumulation of SOX2^+^ cells at the injury epicenter in the SRC group (Fig. 3H). This suggests that SRCs may enhance repair partly by activating intrinsic neuroregenerative mechanisms [35, 36].

In summary, SRCs orchestrate a multi-faceted reparative program following SCI, which includes constraining the lesion area, protecting imperiled neurons in the peri-lesion zone, promoting an axonal growth-supportive glial architecture, and potentially activating endogenous progenitor cells to contribute to neural repair.

### SRC-derived exosomes mediate neuroprotection and functional recovery post SCI

**Figure 4.**
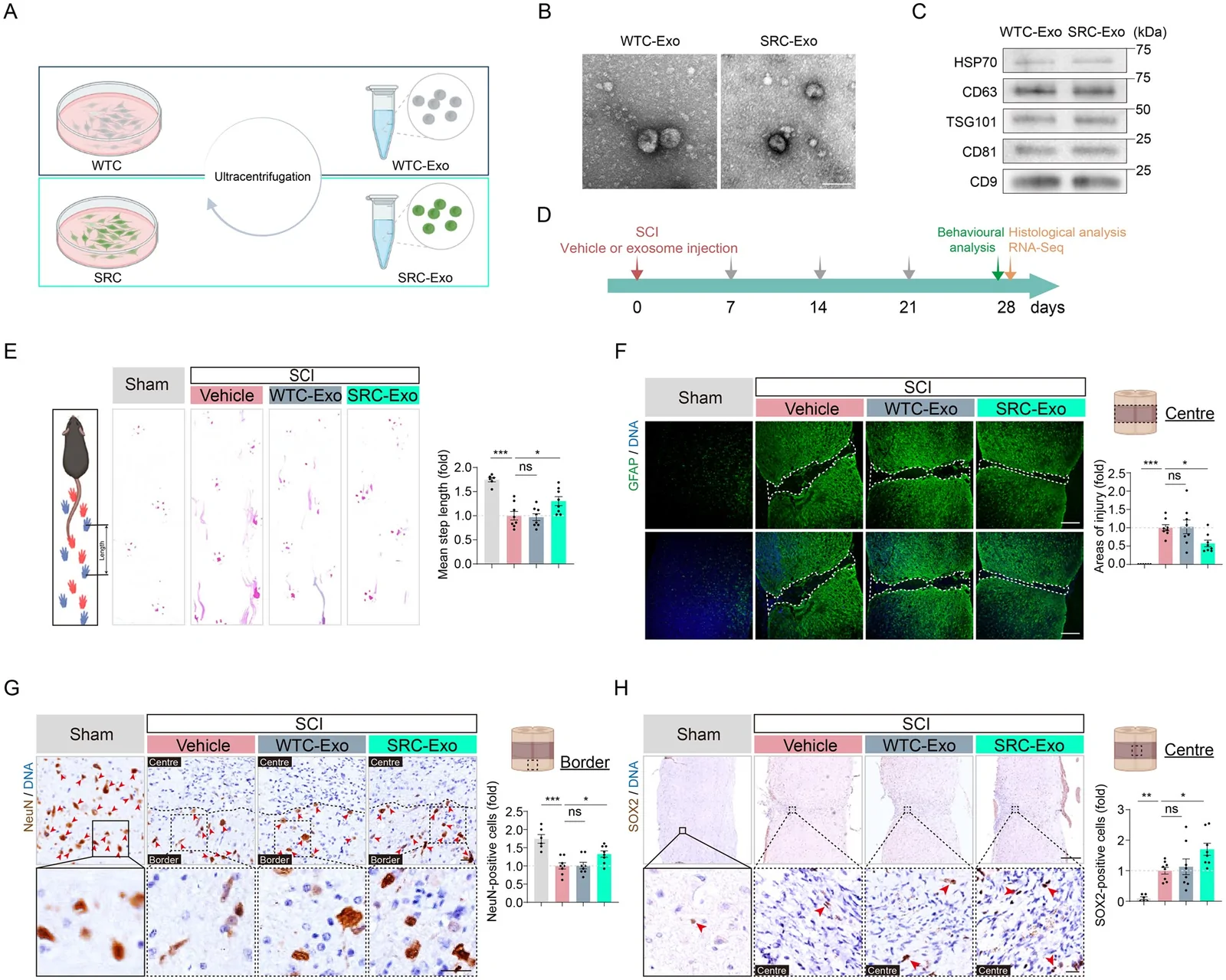
SRC-Exo treatment facilitates functional recovery after SCI.(A) Schematic representation diagram of the exosomes collection. (B) Representative images of analyzed WTC-Exo and SRC-Exo under transmission electron microscope. (C) Western blot analysis of exosome signature markers in WTC-Exo and SRC-Exo. (D) Schematic representation of the experimental design, including animal grouping, the timeline of exosome injection and subsequent behavior, histological and transcriptomic analyses. (E) Footprint analysis and statistical data across indicated groups. (F) Immunostaining analysis of GFAP in spinal cord of mice across indicated groups. White dashed lines outline the area of the injury center. (G) Immunostaining analysis of NeuN in spinal cord of mice across indicated groups. (H) Immunostaining analysis of SOX2 in spinal cord of mice across indicated groups. Quantitative data are shown as means ± SEMs. *n =* 6–8 biological samples per group in (E)–(H). Dashed lines indicate the injury border in (G)–(H); solid and dashed squares indicate the zoom-in areas in (G) and (H). One-way ANOVA followed by Dunnett’s multiple comparisons test was performed in (E)–(H). Scale bar, 100 nm in (B), 20 μm in (G) and lower panel of (H), 200 μm in (F) and upper panel of (H).

Previous studies have demonstrated that exosomes, as key effectors of the therapeutic actions of cell therapy, play a crucial role in intercellular communication and functional regulation [14, 37–39]. Based on these findings, we further explored the therapeutic potential of SRC-Exo. Exosomes were successfully isolated from conditioned media using ultracentrifugation (Fig. 4A). Transmission electron microscopy revealed that both WTC-Exo and SRC-Exo exhibited the typical cup-shaped morphology with a diameter of approximately 100 nm (Fig. 4B). Western blot analysis confirmed the expression of characteristic exosomal markers, including HSP70, CD63, TSG101, CD81, and CD9, in both groups of exosomes [40] (Fig. 4C).

To evaluate the therapeutic effects of exosomes, we used stereotactic techniques to precisely inject exosomes into the site of SCI (Fig. 4D). Behavioral assessments showed that, compared to the vehicle group, the SRC-Exo treatment group exhibited an improvement in motor function at 28 days post-treatment, as evidenced by increased average stride length (Fig. 4E). Histological analyses further revealed that SRC-Exo partially recapitulated the therapeutic effects of SRC implantation by: (1) reducing the lesion area (Fig. 4F); (2) mitigating neuronal loss in the injury border zone (Fig. 4G); and (3) promoting the recruitment of SOX2^+^ cells to the injury epicenter (Fig. 4H). These results indicate that the therapeutic effects of SRCs are partially mediated by the exosomes they secrete, providing evidence for the development of cell-free therapeutic strategies.

### SRC-derived exosomes reprogram the SCI-associated transcriptome

**Figure 5.**
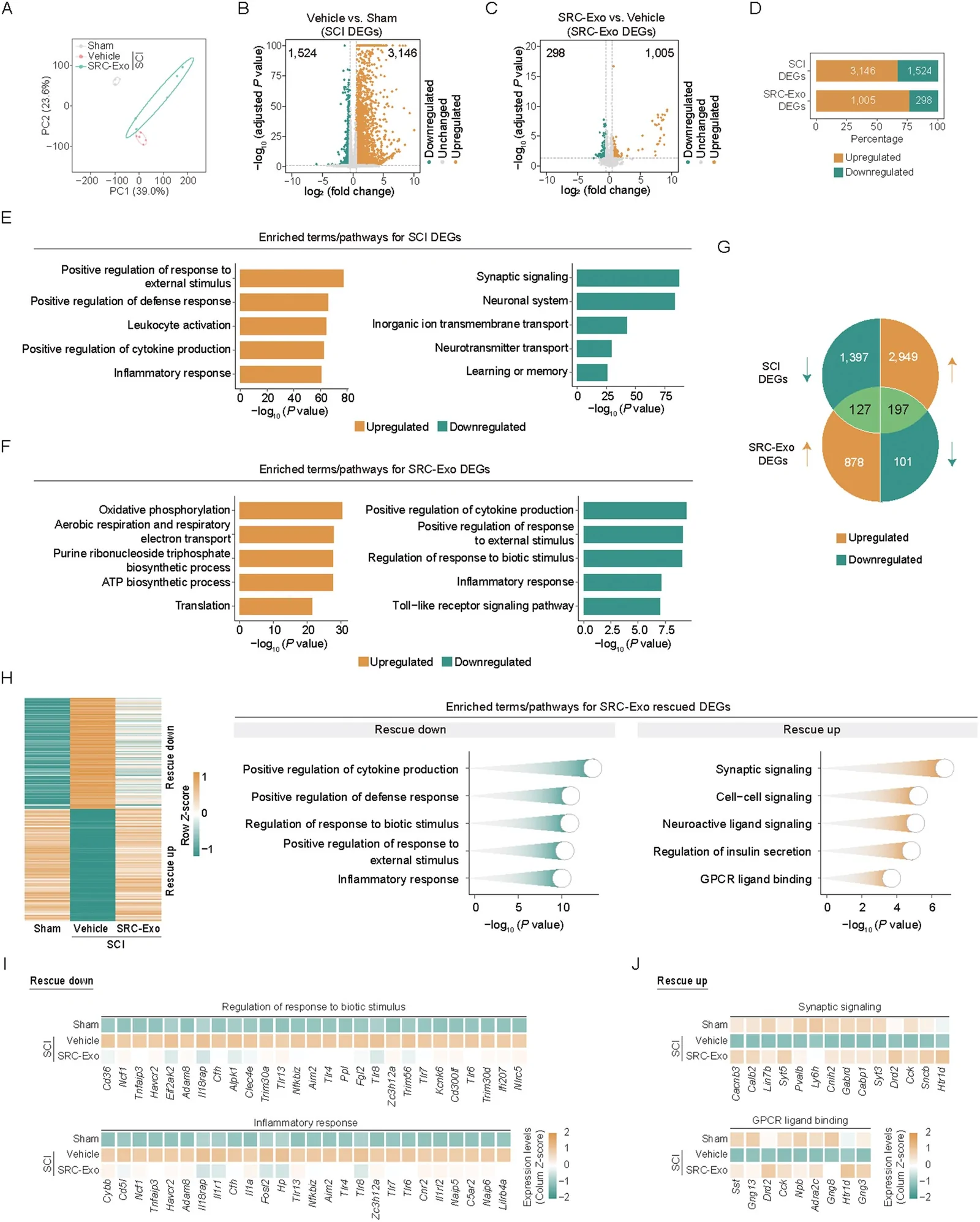
SRC-Exo treatment resets SCI-associated transcriptional profile.(A) Principal component (PC) analysis was performed on RNA-seq data from mouse spinal cord in sham group and in vehicle and SRC-Exo groups after SCI. (B) Volcano plot showing the DEGs between vehicle-treated and sham groups (referred to as SCI DEGs). (C) Volcano plot showing the DEGs between SRC-Exo-treated and vehicle-treated groups (referred to as SRC-Exo DEGs). (D) Bar plot showing the numbers and proportions of upregulated and downregulated SCI DEGs, or SRC-Exo DEGs in the mouse spinal cords. (E) Representative enriched terms and pathways for SCI DEGs in the mouse spinal cords. (F) Representative enriched terms and pathways for SRC-Exo DEGs in the mouse spinal cords. (G) Venn diagram showing the overlapping of SCI DEGs and SRC-Exo DEGs in the mouse spinal cords. (H) RNA-seq analysis of mouse spinal cord in sham, vehicle and SRC-Exo groups. Left, heatmap showing rescued DEGs in the spinal cord of SRC-Exo-treated mice. Right, representative enriched terms and pathways for rescued DEGs in the spinal cord of SRC-Exo-treated mice. (I) Heatmaps showing the relative expression levels of rescued DEGs (downregulated) related to indicated terms in the mouse spinal cords. (J) Heatmaps showing the relative expression levels of rescued DEGs (upregulated) related to indicated terms in the mouse spinal cords.

To elucidate the molecular mechanisms underlying SRC-Exo-mediated SCI repair, we performed RNA sequencing on spinal cord tissues from sham, vehicle-treated, and SRC-Exo-treated groups. Principal component analysis revealed that SRC-Exo treatment partially reversed the SCI-induced transcriptomic perturbations, shifting the gene expression profile toward the sham-operated state (Fig. 5A;  Table S3). Differential expression analysis identified that SRC-Exo rescued 8.33% of SCI-specific upregulated genes, referred to as rescue-down genes, which are primarily involved in immune regulation and inflammatory responses, and 6.26% of SCI-specific downregulated genes, referred to as rescue-up genes, which are mainly enriched in synaptic signaling and G protein-coupled receptors (GPCR) pathways (Fig. 5B–H). Notably, SRC-Exo treatment resulted in the downregulation of the immune-activation-related gene *Tlr8* (Fig. 5I). As a known regulator of the NF-κB pathway, *Tlr8* suppression likely contributes to reduced neuroinflammation [41–44]. Conversely, SRC-Exo treatment upregulated the neurotransmitter-related genes *Gabrd* and *Cabp1* (Fig. 5J). These genes facilitate neural signaling by modulating ion channel activity [45, 46]. These findings, at the transcriptomic level, reveal that SRC-Exo promotes SCI repair through a dual regulatory mechanism: attenuating neuroinflammation and enhancing neurotransmission-related gene expression to facilitate neural function reconstruction, thereby providing a molecular basis for the efficacy of SRC-Exo intervention.

## Discussion

**Figure 6.**
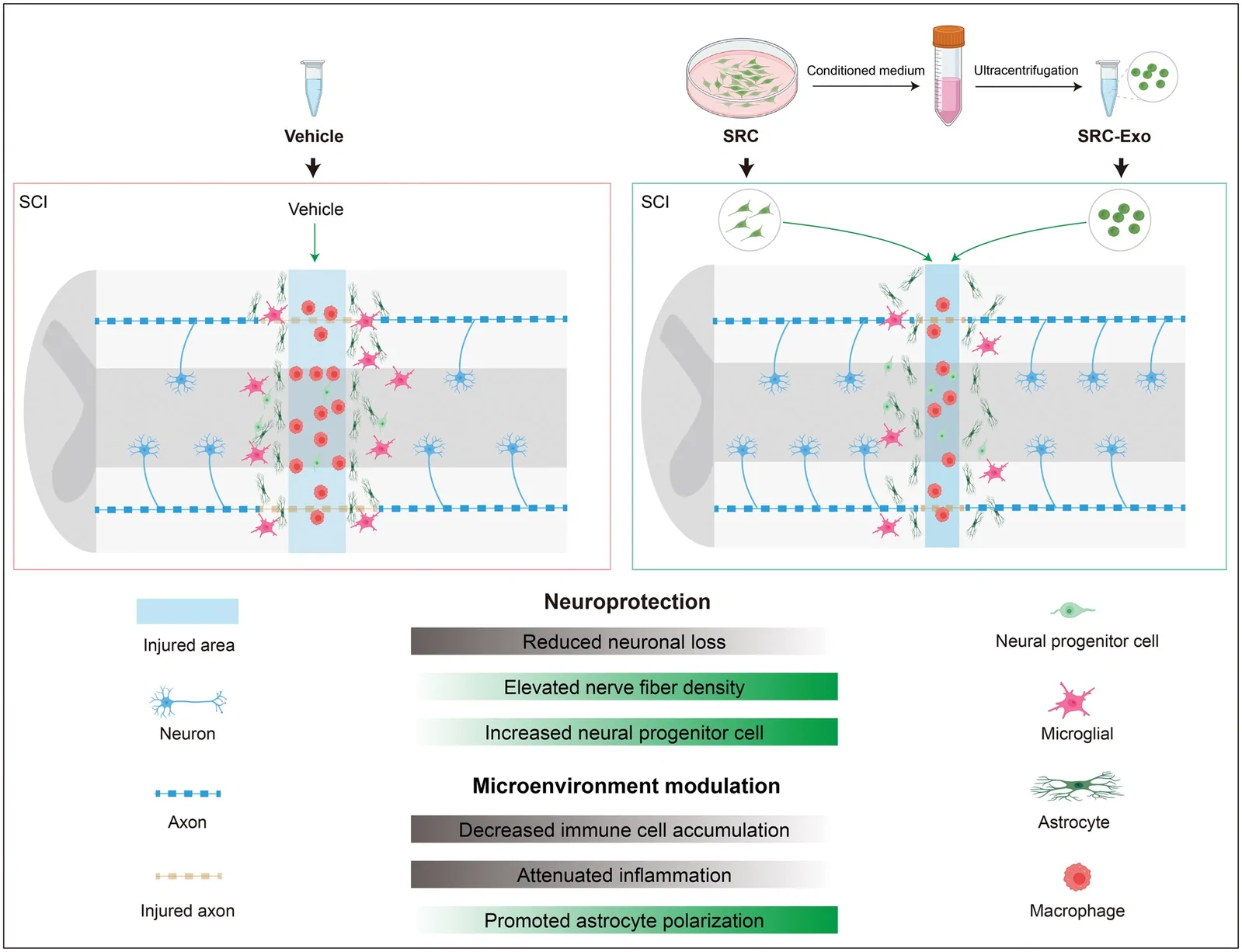
Schematic representation of the mechanism of SRC and SRC-Exo intervention in SCI.

The spinal cord plays a pivotal role in transmitting information between the CNS and peripheral tissues and organs. SCI not only interrupts central neural conduction, leading to loss of motor function and autonomic dysfunction below the level of injury, but also triggers pathological pain and inflammatory responses, causing long-term suffering for patients. Through evaluations at different time points following SCI [47–49], our study demonstrates that genetically engineered SRCs confer therapeutic benefits for SCI repair. Compared to WTCs, the implantation of SRCs improves motor function in mice with SCI. However, due to limitations in the available detection methods in rodents [50], we were unable to assess potential improvements in sensory nervous system function or autonomic nerve innervation in this study. In-depth mechanistic studies reveal that SRCs may promote neural repair through the following synergistic actions: (1) direct neuroprotective effects: reducing neuron loss and axonal degeneration, while activating the endogenous neurogeneration; (2) microenvironment remodeling: modulating neuroinflammatory responses and astrocyte morphological polarization to establish a regeneration-conducive niche (Fig. 6). Of note, our study demonstrated that the therapeutic effects of SRCs are partly mediated by their secreted exosomes. These findings not only expand our understanding of the mechanisms of stem/progenitor cell therapy but also provide two potential strategies for SCI clinical treatment: cell transplantation-based therapy and exosome-based cell-free therapy, both of which hold translational medical value.

Mesenchymal stem/progenitor cell (MSC/MPC)-derived cell therapies are widely investigated due to their core advantages—such as multilineage differentiation potential and homing effects [51, 52]. However, their clinical translation faces challenges including lengthy preparation cycles, batch-to-batch variability, and safety concerns [53]. To overcome these limitations, we developed genetically engineered SRCs designed to enhance neuroprotective efficacy under severe pathological conditions such as SCI. Additionally, exosomes have emerged as a promising cell-free therapeutic strategy, exhibiting translational potential due to their low immunogenicity, ability to cross biological barriers, and ease of administration [40, 54–57]. Consistent with these properties, we observed that SRC-Exo downregulated inflammation-related genes while upregulating genes associated with neurite outgrowth and signal transduction. This aligns with prior studies indicating that exosomes from MSCs/MPCs modulate inflammatory responses and facilitate intercellular communication and functional regulation via bioactive molecules (e.g. proteins and nucleic acids [14, 37]). Together, our findings provide experimental support for both cell-based and cell-free exosome therapies, offering valuable insights for developing advanced clinical treatments for SCI.

Notably, we observed a substantial aggregation of SOX2-positive neural stem/progenitor cells in the injury epicenter, and SRC implantation further increased their abundance. Previous studies have shown that these cells may originate from the ependymal zone of the spinal cord [58, 59], and possess the potential for multilineage differentiation into neurons, oligodendrocytes, and astrocytes [60]. The increase in their numbers may have facilitated endogenous neuroregeneration. Given the reported enrichment of chemokines in exosomes [61], we speculate that SRC-Exo may recruit these progenitor cells via their chemokine content, thereby promoting their migration and proliferation. Future research should focus on elucidating the precise origin of SOX2-positive cells, their migratory routes, differentiation trajectories, and the specific exosomal chemokines involved in this process.

In summary, our study reveals the therapeutic potential of SRCs and their exosomes in SCI repair, thereby providing a foundation for developing advanced treatments for SCI.

### Research limitation

Our study establishes that genetically engineered SRCs promote SCI repair in mice, an effect likely mediated by exosomes. It should be noted that this study has certain limitations. The precise molecular mechanisms remain elusive, as the specific anti-inflammatory and neuroprotective factors within the exosomes have not been identified. Furthermore, the clinical relevance is constrained by the reliance on a mouse model, with known interspecies differences in CNS biology thereby underscoring the necessity of future validation in non-human primates. Despite these limitations, this work lays a promising foundation for stem cell-based neuroregeneration.

## Methods

### Research ethics

All experimental procedures were conducted in accordance with the Guidelines for the Care and Use of Laboratory Animals and in compliance with animal ethics (IOZ-IACUC-2024-279). These experimental protocols were approved by the Institute of Zoology, Chinese Academy of Sciences.

### Generation and characterization of MPCs

Differentiation of hESCs towards hMPCs was performed as described in an established protocol [14, 62]. Briefly, embryoid bodies obtained from hESCs were cultured in MPC differentiation medium consisting of mesenchymal stem cell basal medium (MSCBM) supplemented with 5% HELIOS UltraGRO-Advanced supplement, 1% penicillin/streptomycin, 6 ng/mL bFGF, 5 ng/mL TGF-β, and 10 ng/mL EGF until fibroblast-like cells emerged. The cells were passaged into vitronectin-coated plates, and cultured in MPC culture medium (MSCBM with 5% HELIOS UltraGRO-Advanced supplement). Cells were collected and purified with MPC-specific surface markers CD73, CD90, and CD105, and the absence of hematopoietic stem cell markers CD34, CD43, and CD45 was confirmed by fluorescence-activated cell sorting (FACS). Antibodies used in this study were listed in Table S1.

### Cell culture and exosome isolation

The culture of MPCs followed previous studies [14]. Briefly, MPCs were cultured in MPC culture medium at 37°C with 5% CO_2_. MPCs at passage 7 were used for cell transplantation and the conditioned medium was collected for exosome isolation. To collect exosomes, the conditioned medium was centrifuged at 500 *g* for 5 min and then filtered through a 0.22 μm membrane to remove cells and cellular debris. After 2 h of ultracentrifugation at 100,000 *g*, 4°C, the supernatant was removed, and the exosomes were resuspended in 1 mL of saline, washed and centrifuged at 100,000 *g* for an additional 2 h at 4°C.

### Genomic DNA extraction and DNA sequencing

DNA sequencing was performed to identify specific editing sites after gene editing in the *FOXO3* gene. Genomic DNA was extracted using the DNeasy Blood & Tissue Kit. Exon 3 of *FOXO3* gene contained two mutation sites was amplified by PCR using the PrimeSTAR DNA Polymerase Kit, and the PCR products were sequenced at Tsingke Biotechnology Co. The primers used in this study were listed in Table S2.

### Luciferase reporter assay

The transcriptional activity of FOXO3 was evaluated using the FHRE-Luc vector. WTCs and SRCs were co-transfected with 1.0 μg of the FHRE-Luc vector and 0.2 μg of the Renilla plasmid using Lipofectamine 3000. After 48 h of transfection, the cells were lysed, and luciferase activity was measured using the Dual Luciferase Assay Kit.

### Clonal expansion assay

Clonal expansion assay was performed as described previously [14]: 3,000 MPCs were seeded in each well of a 12-well plate and cultured for 10 days. Cells were then fixed and stained with crystal violet. The cell density was calculated using ImageJ.

### SA-β-gal staining

SA-β-gal staining was performed as described previously [63–65]. Briefly, MPCs were fixed in fixation buffer (2% formaldehyde and 0.2% glutaraldehyde) for 5 min, followed by washing three times with PBS and staining by SA-β-gal staining solution at 37°C for 8 h. The percentage of SA-β-gal-positive cells was quantified using ImageJ.

### Telomere length analysis

Quantitative PCR (qPCR) was used to quantify telomere length, as detailed previously [14]. The primers used in this study are listed in Table S2.

### Cell immunofluorescence staining

Cell immunofluorescence staining was performed according to the previous publication [66]: 4% paraformaldehyde (PFA)-fixed cells were permeabilized with 0.4% Triton X-100 for 10 min, washed three times with PBS for 5 min each, blocked with 5% donkey serum at room temperature for 1 h, washed three times with PBS for 5 min each, incubated with primary antibody overnight at 4°C, washed three times with PBS for 5 min each, incubated with secondary antibody and Hoechst 33342, washed with PBS, and mounted with anti-fade agent. Ultimately, the images were captured using the Zeiss LSM 900 confocal microscope system and analysis of the data was performed by ImageJ. Antibodies used in this study were listed in Table S1.

### Western blot

Western blot was performed according to the previous publication [67]. In brief, well-prepared protein samples were subjected to electrophoresis, membrane transfer, and antibody incubation, and then imaged using the ChemiDoc XRS system (Bio-Rad) and analysis of the data was performed by ImageJ. Antibodies used in this study were listed in Table S1.

### Cell cycle analysis

Cell cycle analysis was performed according to the previous publication [68]. In brief, cells were collected and fixed with pre-chilled 70% ethanol overnight at −20°C. The cells were then washed once with PBS and incubated at 37°C for 30 min in PBS containing 0.1% Triton X-100, 0.2 mg/mL RNase A, and 0.02 mg/mL propidium iodide solution. The cells were tested directly after incubation using an LSRFortessa cell analyser (BD) and analyzed using ModFit software.

### Animal experiments

The study utilized 8-week-old male C57BL/6J mice (SPF Biotech Co., Ltd) that were maintained under controlled environmental conditions (25°C, 50%–60% humidity) with a 12:12-hour light–dark cycle. Animals were provided with standard laboratory diet and water *ad libitum* throughout the acclimatization and experimental periods.

#### Establishment of mouse SCI model

Eight-week-old male C57BL/6J mice were used to establish a SCI model [21]. Following anesthesia with isoflurane, the dorsal hair was shaved and the skin was disinfected before a 1-cm incision was made in the back skin with scissors. Following muscle retraction, a T10 laminectomy was performed to expose the underlying spinal cord without dural disruption. SCI was induced by applying a vascular clip for 2 s at the T10 level. The sham group mice underwent identical surgical procedures, including laminectomy, but without spinal cord compression. The skin was sutured, disinfected with iodophor, and the mice were released into the cage after being awake to promote the mice auto-urination function. Postoperative care included manual bladder expression twice daily until reflex voiding recovered.

#### Cell implantation and exosome injection

After the model was successfully established, 1 μL of saline (as a vehicle) or 1 μL of saline containing WTCs (1 × 10^5^ cells) or SRCs (1 × 10^5^ cells) was delivered using a micro stereotactic injection device, three injection sites including the injury center, 1 mm rostral and 1 mm caudal of the injury center were selected for injection. The depth of injection was 0.75 mm, and a tipless insulin needle was used. For exosome injection, the total protein concentration of exosome was 500 μg/mL, the injection dose was 1 μL, and the operation was the same as the cell injection procedure.

#### *In vivo* bioluminescence imaging

The retention of transplanted MPCs was assessed by longitudinal *in vivo* bioluminescence imaging [16]. In brief, MPCs were transduced with a luciferase-expressing lentiviral vector prior to implantation into the spinal cord. Bioluminescent signals were then quantified using an IVIS Lumina XRMS Series III system at scheduled time points from day 0 to day 7 post-SCI.

#### Footprint analysis

In order to obtain the footprints of the mice, we applied red and blue ink to their forelimbs and hindlimbs, respectively, as markers. As the mice moved forward, their movement trajectories were clearly recorded on the paper. The resulting footprints were then used to measure the stride length and width.

### Histological analysis

Mice were anaesthetized and perfused by saline via the heart. Collected spinal cord samples were fixed by 4% PFA at 4°C overnight, dehydrated with a gradient of alcohol and xylene and embedded in paraffin. After embedding, the spinal cord was cut into 5-μm-thick slices longitudinally with a paraffin slicer. After dewaxing and rehydration, the slices were subjected to high-temperature antigen retrieval using citric acid, and then washed with PBS three times after cooling naturally; the slices could then be used for immunostaining.

Immunofluorescence staining was performed as described previously [69]. Spinal cord sections were permeabilized with 0.4% Triton X-100 for 30 min and then blocked with 5% BSA for 1 h at room temperature. Subsequently, the sections were incubated with the primary antibody overnight at 4°C. The next day, sections were washed with PBS three times, incubated with a fluorescence-labeled secondary antibody for 1 h at room temperature, and washed again with PBS three times. Finally, nuclei were counterstained with Hoechst 33342, sections were mounted with an antifade medium, and images were acquired using a Zeiss LM980 microscope.

For immunohistochemical staining, sections were first subjected to endogenous peroxidase quenching, then permeabilized with 0.4% Triton X-100 for 30 min and blocked with 5% BSA for 1 h at room temperature. They were then incubated with the primary antibody overnight at 4°C. After washing with PBS three times, sections were incubated with a histochemistry-compatible secondary antibody for 1 h. Following another round of PBS washes, color development was performed using diaminobenzidine (DAB) reagent. Subsequently, nuclei were stained with hematoxylin, after which the sections were dehydrated through a graded ethanol series (70%, 95%, and 100%) and xylene (two changes, 5 min each). Finally, sections were mounted with a neutral mounting medium and imaged with a Nikon microscope.

All image analysis was performed using ImageJ. Antibodies used in this study are listed in Table S1.

### RNA-seq data analysis

In the RNA-seq analysis of mouse data, raw sequencing reads were first processed using Trim Galore (v.0.6.7) to remove low-quality bases and adapter sequences. Subsequently, the cleaned reads were aligned to the mm10 mouse reference genome obtained from the Ensembl genome browser using the STAR (v.2.7.1a) [70]. The number of reads mapped to each gene was quantified using featureCounts (v.2.0.1) [71]. Differential expression analysis was performed using DESeq2 (v.1.30.1) [72], with differentially expressed genes (DEGs) identified based on a criterion of an adjusted *P* value < 0.05 and an absolute log_2_ (fold change) > 0.5. Functional enrichment analysis of the identified DEGs was conducted using Metascape [73] to elucidate Gene Ontology terms and pathways. The complete list of DEGs is provided in Table S3.

### Statistical analysis

Two-sided Student’s *t* test, Wilcoxon rank-sum test, and one-way ANOVA followed by Dunnett’s multiple comparisons test were used in this study. Statistical significance was defined as *P *< 0.05. Significance levels were denoted as follows: *P < 0.05, **P < 0.01, ***P < 0.001, with “ns” indicating non-significant results.

## Supplementary Material

lnaf038_Supplementary_Data

## Data Availability

The RNA-seq data underlying this article have been submitted to the Genome Sequence Archive [74] in the National Genomics Data Center [75], Beijing Institute of Genomics (China National Center for Bioinformation) of the Chinese Academy of Sciences, with accession number CRA027637.
